# Prevalence and risk factors of posttraumatic stress disorder among teachers 3 months after the Lushan earthquake: A cross-sectional study

**DOI:** 10.1097/MD.0000000000004298

**Published:** 2016-07-22

**Authors:** Jun Zhang, Ye Zhang, Changhui Du, Shenyue Zhu, Yalin Huang, Yulian Tian, Decao Chen, Haimin Li, Yao Gong, Mengmeng Zhang, Bo Gu

**Affiliations:** a Mental Health Center, West China Hospital, Sichuan University, Chengdu, China; b Education Supervision Department, Baoxing County Education Bureau, Yaan, China; c Science and Education Information Department, Chengdu Center of Disease Control, Chengdu, China; d People’ Hospital of Yaan, China; e Department of Nursing, West China Hospital, Sichuan University, China.

**Keywords:** earthquake, posttraumatic stress disorder (PTSD), prevalence, risk factors, teacher

## Abstract

Teachers and students often suffer from the same disaster. The prevalence of PTSD in students has been given great attention. However, in acting as mentors to students and their families, teachers are more likely to have vicarious and indirect exposure via hearing stories of their aftermath and witnessing the consequences of traumatic events. There are limited data pertaining to the prevalence of PTSD and its risk factors among teachers. A total of 316 teachers from 21 primary and secondary schools in Baoxing County were administered a project-developed questionnaire which included the items regarding demographic characteristics, earthquake-related experiences, somatic discomforts, emotional reactions, support status, and everyday functioning 2 weeks after the Lushan earthquake, and they finished a 1-to-1 telephone interview for addressing the PTSD criteria of the Mini International Neuropsychiatric Interview (MINI) 3 months after the earthquake. The prevalence of PTSD was 24.4% among teachers. Somatic discomforts (odds ratio [OR] 1.89, 95% confidence interval [CI] 1.06–3.37) were positive risk factors of PTSD. Perceived social support (OR 0.30, 95% CI 0.14–0.62) and being able to calm down (OR 0.25, 95% CI 0.09–0.75) in teaching were negative risk factors. PTSD is commonly seen among teachers after an earthquake, and risk factors of PTSD were identified. These findings may help those providing psychological health programs to find the teachers who are at high risk of PTSD in schools after an earthquake in China.

## 1 Introduction

On April 20, 2013, a 7.0 Richter-scale earthquake erupted in Sichuan province, China. The epicenter was located in the Lushan County, which was also affected by the Wenchuan Earthquake in 2008.^[1]^ Only 5 years separated these 2 catastrophic earthquakes. The Lushan earthquake resulted in 196 deaths, and at least 11,470 were injured, with more than 968 seriously injured. A large number of houses and buildings were destroyed, forcing many local inhabitants to live in temporary settlements. In addition, psychological changes have been reported among survivors.^[2]^

Posttraumatic stress disorder (PTSD) is the most common psychological sequelae in adult survivors after an earthquake.^[3–5]^ For example, 6 months after the Wenchuan earthquake, the prevalence of PTSD was 15.6% among 14,207 individuals from Dujiangyan, Beichuan County, and Qingchuan County.^[6]^ Additionally, the prevalence of PTSD was 19.2% in a randomly selected sample consisting of 683 survivors 3 years after the Marmara earthquake.^[7]^ The prevalence of PTSD in students has also been given great attention.^[8–12]^ When it comes to students’ mental health after a disaster, teachers are responsible for the acute emotional, physical, and informational needs of students once they understand the role as educators.^[13,14]^ To be effective as both caregivers and educators for students, teachers’ own mental health conditions should be addressed. Although students and teachers may suffer from the same disaster, there are limited data pertaining to the prevalence of PTSD in teachers.

When seeking to identify PTSD symptoms in adults after an earthquake, several identified risk factors of PTSD, such as age,^[6]^ sex,^[15]^ ethnicity,^[15]^ social support,^[16,17]^ death of family members,^[3]^ witnessing someone being killed,^[17]^ damaged household,^[3]^ and living in a shelter or temporary house^[5]^ should be considered. The majority of previous studies explored the risk factors of PTSD largely in isolation from other putative risk factors, such as sleep problems^[18,19]^ and somatic discomforts,^[20,21]^ which have been well-documented. Everyday functional impairment was also found to be related to psychological trauma,^[22,23]^ because small changes of everyday functioning can be predictive of an increased risk for future pathologic behavior and be a source of psychological distress.^[24,25]^ To our knowledge, there is no study included all the aforementioned variables to explore the risk factors of PTSD, let alone in teachers.

These findings in previous studies have implications for preventing PTSD among survivors after earthquakes. For those providing psychosocial interventions, educations and interventions could be used to help survivors to deal with PTSD. Although many studies have focused on PTSD, its prevalence and risk factors among teachers after an earthquake are still unclear. We hypothesized that PTSD symptoms were commonly seen in teachers 3 months after the Lushan earthquake. In addition, we hypothesized that risk factors such as sex, age, somatic discomforts, and the everyday functioning were associated with PTSD in teachers. Therefore, the aims of the present study were to explore the prevalence and risk factors of PTSD in teachers 3 months after the Lushan earthquake in China.

## 2 Methods

### 2.1 Ethical considerations

This study was approved by the Research Ethics Committee of the West China Hospital of Sichuan University, the Department of Health of Sichuan Province, and the Education Bureau of the Baoxing County. Written informed consent was obtained from each school principal. Before conducting this investigation, investigators gave teachers a description of the procedures and informed them that they could participate in this study voluntarily and that they could withdraw from this study. Written informed consent was obtained from each teacher in the questionnaire.

### 2.2 Participants and procedure

The present study was conducted in Baoxing County, which was one of the most severely damaged counties during the earthquake. There are 21 primary and secondary schools in Baoxing. It was selected because the schools’ principals and the manager of Education Bureau of Baoxing County were aware that it is important to perform a survey regarding the psychological conditions which could benefit the psychosocial intervention in teachers, and they were willing to provide assistance for our study.

Participants of this study underwent a 2-stage assessment. The data of the first stage were collected at the beginning of May 2013, about 2 weeks after the Lushan earthquake, in which participants should finished a project-developed questionnaire under the supervision of trained individuals with a master's degree in psychology. The data of the second-stage were collected at the end of July 2013, more than 3 months after the earthquake, in which participants should finished a 1-to-1 telephone interview for addressing the PTSD criteria of the Mini International Neuropsychiatric Interview (MINI) by 4 investigators who had received training in the use of diagnostic tools. To increase the investigator consistency, during all interviews, an additional investigator listened to the conversion between the primary investigator and the participant, and the diagnosis of both investigators was compared. If investigators meet an inconsistent diagnosis, they will go to discuss with a professor working in psychiatry department who can help investigators to meet the consistent diagnosis. In the first stage, a sample of 506 participants was recruited from the 21 primary and secondary schools in Baoxing County. The inclusion criterion was willingness to participate, and the exclusion criteria were refusal to participate and failure to complete the majority of the investigation. Of the possible 506 participants, no participant was excluded in the first stage. In the second stage, 190 participants who refused to answer the telephone or who could not finish the telephone interview or who refused to participate in the telephone interview were excluded. Finally, data for 316 teachers were complete and suitable for analysis.

### 2.3 Measures

We used a project-developed questionnaire to estimate the variables regarding the demographic characteristics including sex, age, marital status, and ethnicity; the earthquake-related experiences including having direct relatives seriously injured in the earthquake, house damage, loss of property seriously; sleep and somatic problems including 3 sleep-related items (i.e., falling asleep difficult, waking up with a start or nightmare, and waking up earlier), increased somatic discomforts, and decreased appetite; emotional reactions including being sad easily, being perturbed easily, and being anxious easily; everyday functioning including being able to calm down in teaching, observing severe inattention of students in class, being able to communicate with colleagues effectively, being able to communicate with family members effectively, perceived social support, and perceived family support. In this study, age was divided into ≤35 years and >35 years. Ethnicity was divided into Han Chinese and minorities. Marital status was divided into married and nonmarried. Other questions were coded into yes/no items.

Prevalence of PTSD was assessed using the MINI 5.0.0, which generates psychiatric diagnoses based on DSM-IV criteria and can be administered by trained personnel or psychiatrists (e.g., nurses, medical doctors, correctional officers, and psychologists).^[26–29]^ The psychometric properties and concordance of the MINI analyzed by reference either to the Structured Clinical Interview for Diagnostic and Statistical Manual (DSM)-III-R (SCID)^[30]^ or to the Composite International Diagnostic Interview (CIDI)^[31]^ have demonstrated a satisfactory level, which could facilitate the application of our study. The MINI Chinese version has shown good validity and reliability in China.^[32,33]^

### 2.4 Statistical analysis

Descriptive statistics were computed for categorical variables. Univariate logistic regression analyses were used to examine the bivariate relationship between PTSD and putative risk factors. Multivariate logistic regression analyses were used to identify positive and negative risk factors of PTSD. The associations between risk factors and PTSD were estimated using odds ratio (OR) with respective 95% confidence intervals (CIs). Statistical analyses and calculations were performed using the Statistical Package for the Social Sciences (SPSS) version 19.0 with 2-tailed *P* values <0.05 considered as statistically significant.

## 3 Results

### 3.1 Investigator reliability of the MINI-PTSD

A total of 40 cases were evaluated in this manner. Three cases presented with inconsistent diagnosis from the 4 investigators resulting in an investigator reliability of 92.5%.

### 3.2 Demographic characteristics

Of the 316 participants, 173 (54.7%) were female, 253 (80.1%) were Han Chinese, and 239 (76.1%) were married (Table 1). Their ages ranged from 20 to 60 years with a mean of 37.28 ± 10.12 years.

**Table 1:**
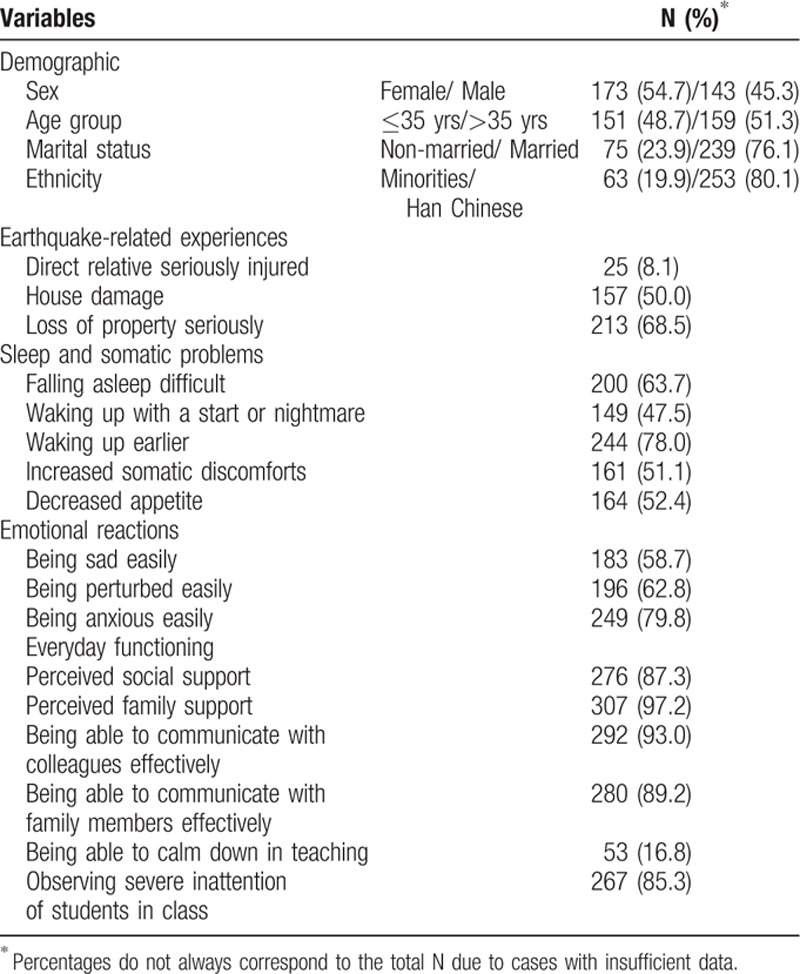
Description of Study Population (N = 316).

### 3.3 Prevalence and risk factors of PTSD among teachers

In the present study, the prevalence of PTSD was 24.4%. Bivariate logistic regression analysis identified several variables that were associated with PTSD (Table 2). A multivariate logistic regression analysis indicated that positive risk factor of PTSD was increased somatic discomforts (OR 1.89, 95%CI 1.06–3.37). Perceived social support (OR 0.30, 95%CI 0.14–0.62) and being able to calm down during teaching were negative risk factors of PTSD (OR 0.25, 95% CI 0.09–0.75) (Table 2).

**Table 2:**
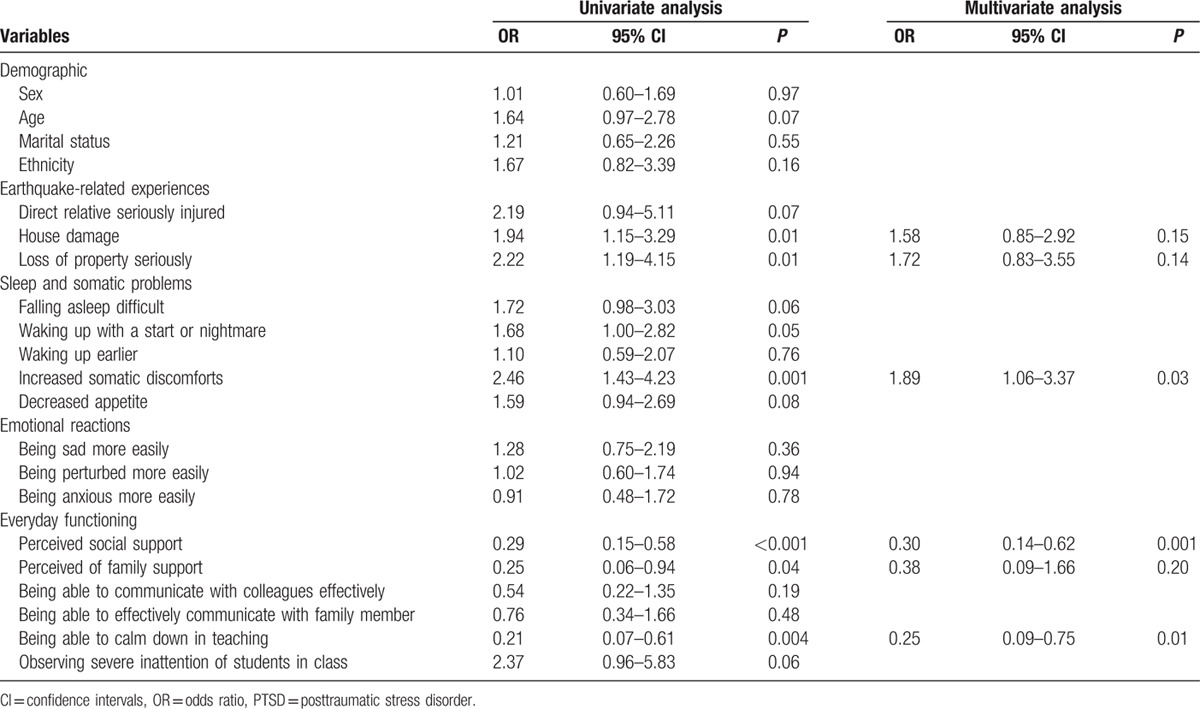
Logistic regression analyses of risk factors of PTSD among teachers.

## 4 Discussion

To the best of our knowledge, the present study is the first research to explore the prevalence of PTSD in teachers after an earthquake. Our results on the prevalence of PTSD are comparable with postdisaster prevalence documented elsewhere. Increased somatic discomforts were positive risk factors significantly related to the subsequent development of PTSD in teachers after the Lushan earthquake. Postdisaster mental health recovery interventions such as early identification, close monitoring, and sustained psychosocial support are necessary for the teachers who are at high risk for PTSD in China.

### 4.1 Prevalence of PTSD

In the present study, the prevalence of PTSD in teachers was 24.4%, which is relatively high compared with previous study conducted among survivors after an earthquake. One study showed that the prevalence of PTSD was 15.57% six months after the Wenchuan earthquake.^[6]^ Additionally, the prevalence rates of PTSD among the Chi-Chi earthquake victims after 6 weeks to 10 months are about 7.9% to 21.7%.^[34–37]^ Teachers in disaster areas are not only vulnerable for direct victimization common to all citizens, but may also face added challenges through their profession.^[38]^ They often focus on reducing the posttraumatic stress symptoms of their students.^[14,39]^ In acting as mentors to students and their families, they are also likely to have vicarious and indirect exposure via hearing stories of their aftermath and witnessing the consequences of traumatic events.^[38]^ These could be partly explained by the relatively high prevalence in our study, and differences in study measurements and assessing times should also be considered.

### 4.2 Risk factors of PTSD

A well-established finding is that female is a risk factor of PTSD after traumatic events,^[40,41]^ which may be explained by the sex differences in brain activation to stress^[42]^ and different coping styles and socioeconomic status of females and males.^[43]^ However, this relationship was not significant in the present study, which is consistent with the findings of Kun et al.^[44]^ This could be explained by the following: the Baoxing County was erupted by the Wenchuan Earthquake in 2008, and the teachers in Baoxing have the experiences to cope with the traumatic events under the help of Chinese government, which might reduce the sex differences of coping styles between males and females after surviving a disaster.

Previous studies have explored the relationship between age and the development of PTSD. For example, Zhou et al^[6]^ reported that the survivors with older age are at risk for the development of PTSD 6 months after the Wenchuan earthquake. Cheng et al^[45]^ also found that older age was significantly associated with PTSD 1 year after the Wenchuan earthquake. However, younger age as one of the risk factors of PTSD has also been reported.^[15,46–49]^ In the present study, our results showed that age was not associated with the development of PTSD, which should be further verified in future.

Regarding somatic discomforts, teachers with an increase in somatic discomforts at 2 weeks were more likely to develop PTSD at 3 months. The association between somatic discomforts and PTSD has also been well-documented in previous studies.^[19,20,50]^ Exploring this relationship is a critical concern due to the demonstrated potential to impact the course of functional disability and psychological distress among survivors with traumatic experiences.^[51]^ Somatic discomforts may delineate a specific pattern of posttraumatic stress symptoms.^[52]^ The findings of the current study suggest that the association between somatic discomforts and the subsequent development of PTSD should be closely monitored in teachers with traumatic experiences.

Teachers who experienced social support had a lower risk for developing PTSD, which is similar to previous studies.^[46,53–55]^ Xu and Song^[15]^ reported that low social support is an important risk factor for the development of PTSD 1 year after the Wenchuan earthquake. The social support may be useful in PTSD intervention and prevention measures for teachers with traumatic experiences in the early stage after an earthquake.

The association between everyday functional impairment and psychological trauma has been well-demonstrated in previous studies.^[22,23]^ Although small changes in everyday functioning might seem insignificant, they can be predictive of an increased risk for future pathologic behavior by an individual and be a source of psychological distress.^[24,25]^ One study including 20 adolescents who later completed suicide and 20 matched living adolescents found that those who later died through suicide experienced greater degrees of difficulty in performing everyday functions.^[25]^ To the best of our knowledge, there is no study to explore whether everyday functional impairment could predict the subsequent development of PTSD. As for teachers, the performance of teaching is an important manifestation of their everyday functioning. In the present study, our results show that being able to calm down in teaching was a negative risk factor of PTSD. This suggests that teachers who are not able to calm down in teaching should be closely monitored, which may help to find the teachers who are at high risk of PTSD.

Several limitations of this study should be mentioned. Firstly, in this study, we used a telephone interview, due to which some useful information for diagnosis may be lost; so it is necessary that face-to-face interviews should be conducted in future studies. Secondly, the participants of this study were a convenient sample residing in Baoxing County. Therefore, it is unclear whether the findings of our study are generalizable to other teachers exposed to the earthquake. Thirdly, data on demographic variables such as family psychiatric illness and history of psychiatric illness were not collected. Finally, the present study only described the prevalence of PTSD in teachers 3 months after the earthquake. Longitudinal studies are necessary to follow up with the changes. Despite these limitations, this study is the first study reporting the prevalence of PTSD and its risk factors in teachers after an earthquake.

In summary, this study indicated that PTSD symptoms were commonly seen. Somatic discomforts could predict PTSD among teachers in schools, whereas teachers with perceived social support and who are able to calm down in teaching are at a lower risk for the development of PTSD. More importantly, these findings may help those providing psychological health interventions to find teachers who are at high risk of PTSD in schools after an earthquake in China.
